# Comprehensive evaluation of loess collapsibility of oil and gas pipeline based on cloud theory

**DOI:** 10.1038/s41598-021-94882-2

**Published:** 2021-07-29

**Authors:** Xiaohui Liu, Manyin Zhang, Zhizhong Sun, Huyuan Zhang, Yimin Zhang

**Affiliations:** 1 School of Civil Engineering and Mechanics, Lanzhou University, Lanzhou, 730000 Gansu China; 2 Institute of Geological Hazards Prevention, Gansu Academy of Sciences, Lanzhou, 730000 Gansu China; 3 Southwest Pipeline Company, Network Group Corporation, Chengdu, 730000 Sichuan China

**Keywords:** Environmental sciences, Natural hazards

## Abstract

The comprehensive evaluation of pipeline loess collapsibility risk is a necessary means to control the safety risks of pipelines in the collapsible loess section. It is also one of the critical scientific bases for risk prevention, control, and management. The comprehensive evaluation system of cloud theory consists of quantitative and qualitative indexes, and the evaluation system has the characteristics of randomness and fuzziness. In view of this problem, the standard qualitative and semi-quantitative evaluation methods have intense subjectivity in dealing with the uncertainty problems such as randomness and fuzziness of the system, the cloud theory, which can effectively reflect the randomness and fuzziness of things at the same time, is introduced. The state scale cloud and index importance weight cloud of pipeline loess collapse risk are constructed by the golden section method. The uncertainty cloud reasoning process of the quantitative indexes and the expert scoring method of the qualitative indexes are proposed. The comprehensive evaluation model of loess collapsibility risk of oil and gas pipeline is established, and the engineering example is analyzed. The complete evaluation results of 10 samples to be evaluated are consistent with the results of the semi-quantitative method and are compatible with the actual situation. The evaluation process softens the subjective division of index boundary, simplifies the preprocessing of index data, realizes the organic integration of quantitative and qualitative decisions, and improves the accuracy, rationality, and visualization of the results.

## Introduction

Oil and gas pipeline geological disasters are refer to the evolution process of geological processes or geological environment that threaten and endanger the safety and operation environment of oil and gas pipeline engineering^1–3^. Geological disasters mainly include landslides, water damage, loess collapsibility, frost heave, thaw settlement, wind erosion sand burial, salt expansion and swelling settlement, etc^4,5^, which are Influenced by differences in geology, landform, meteorology, and other environmental differences along the onshore long-distance pipeline, respectively. Collapsible loess disaster of the pipeline is a kind of geological disaster which is easy to occur and frequently occurs. It seriously affects the safe and green operation of the pipeline project laid in the collapsible loess stratum^2,3^. The loess plateau of China, where the collapsible loess strata are connected in great thickness, is where the national gas (oil) is transported from the west to the east and the oil (gas) from the north to the south. So far, the total mileage of pipelines in service in this area is more than 5000 km. More than 80% of the various geological disasters suffered along these lines are related to loess collapsibility. The risk of pipeline loess collapsibility is often used to characterize the line's possibility and degree of damage. Scientific and reasonable analysis and evaluation of its status are necessary means to control the safety risk of the line, and it is also the critical scientific basis for disaster monitoring, governance, and management^6–8^.

At present, domestic and foreign scholars have conducted a lot of studies on pipeline geological disaster risk assessment^1,2,7–14^. For example, Tong et al. comprehensively identified and evaluated the possible risk factors of long-distance pipelines based on expert consulting scoring method and system safety theory^9^. However, in this study, as well as in literature 2 and 7, the identified impact factors are mainly assigned by expert rating, and the risk results are obtained by simple mathematical linear operation. The evaluation process ignored the randomness, fuzziness, and other complex problems of the risk system, so the accuracy and scientificity of the results need to be improved. Jamshidi et al.^10^ established a pipeline comprehensive risk assessment model based on fuzzy logic and relative risk scoring by combining qualitative and quantitative evaluation. Fayaz et al.^11^ established a heuristic membership function hierarchy fuzzy reasoning model and applied it in the risk assessment of water supply pipelines. Jamshidi and Fayaz et al. put forward the risk of qualitative and quantitative indicators based on fuzzy membership inference based on the complexity and comprehensibility of the evaluation system. The risk assessment method provides a good idea for the quantitative assessment of the complex system of pipeline geological hazard risk. However, there is still intense subjectivity in the division of the evaluation target and index scale, and the randomness of the system is ignored. There are also cumbersome and time-consuming procedures in the operation process, such as the unified processing of the index dimension. Wang et al.^12^ developed a landslide risk assessment system for the long-distance pipeline based on the expert system platform (ES) by analyzing a large number of landslide cases in China's West-to-East Gas Pipeline. This method relies too much on the existing knowledge base and experience judgement. Its application scope and objects are generally strictly limited, and the accuracy of the assessment results is not high. In addition, the late knowledge updating and maintenance of the system platform are of significant workload and high cost. Vasseghi et al.^13^ used the finite element analysis (FEA) method to evaluate the response relationship of pipelines to ground displacement in the potential landslide area, and combined with theoretical analysis, revealed that the main reason for bending failure of pipelines was the high shear stress at the girth weld. Although numerical simulation analysis methods have high accuracy, its simulation boundary assumptions for monomer disasters complex variability among different location, geological, environmental conditions, and simulate the required parameters in general still need using soil mechanics test. Therefore this method does not apply to the universal development of the pipeline in loess collapsibility disaster danger evaluation and sorting work quickly. Teng et al.^14^ used earthquake scenario analysis and spatial network method to classify and evaluate the seismic damage of urban underground pipelines. It proposed a pipeline disaster impact assessment model based on a geographic information platform. As a result, the form and results can be better grasped. It also predicts the susceptibility of geological disasters along the route to the division and segmentation. It visually displays the relevant developments in the form of maps, but it is not suitable for evaluating the risk status of individual geological disasters. In addition, the researches on loess subsidence disasters of oil and gas pipelines in existing works of literature primarily focus on the analysis of the influencing factors and development mechanism of the classification of uneven settlement types in loess caves and gullies along the pipelines in the loess region^5,15–19^. There are also studies on mechanical behaviors and monitoring and early warning of stress–strain subsidence and suspension of pipelines based on the tubing-soil coupling model or elastoplastic foundation model^19–24^. Yu et al.^25^ evaluated the hazard of loess collapse using the fuzzy mathematics method based on the investigation of pipeline geological hazards. Gao et al.^26^ based on the principle of coupling synergy (CCP), evaluated the pipeline loess collapsibility risk from two subsystems of soil and pipe body and provided better guidance for engineering practice. Yang^27^ and Zhou^28^ established a fuzzy comprehensive evaluation model based on field investigation and hazard evaluation analysis of pipeline loess collapse disasters and applied it to different pipe sections in loess areas. The overall effect was better than the subjective qualitative evaluation results. On the other hand, the above-related studies^15–28^, on the one hand, paid little attention to the risk analysis and evaluation of the pipeline loess collapsibility disaster with universal development. The disaster-bearing environment's comprehensive factor analysis of the disaster-bearing capacity of the disaster-causing factors was not systematic and in-depth. In addition, the selection of indicators was not extensive. On the other hand, index data acquisition and processing showed more complicated time-consuming and continuity of quantitative and qualitative discrete data fusion. Participate in the evaluation process of subjectivity is strong, the fuzziness and randomness model for the complex system in operation to consider problems of one sector. Therefore, it is necessary to continuously optimize and introduce new ideas and propose new methods based on previous studies to provide diversified and high-quality scientific and technological support and services for the risk assessment of pipeline loess collapsibility disasters.

Pipeline loess collapsibility risk is a system composed of loess collapsibility in laying area and pipeline vulnerability, affected by many factors^15,29^. Its evaluation organization is characterized by multiple attributes, multiple levels, and multiple indicators and has both quantitative and qualitative indicators. It is the key to determining the success or failure of the evaluation and the accuracy of integrating these uncertain indicators, which have randomness, fuzziness, and different magnitude and dimension, and participate in the risk evaluation. Cloud theory^30^ is often used to describe the relationships between qualitative and quantitative uncertainty things into a powerful mathematical tool, integrates the advantage of fuzzy mathematics and probability statistics, can effectively reflect the properties of things at the same time the randomness and fuzziness, and constitute a mapping between qualitative and quantitative, makes the qualitative concept and quantitative numerical transformation between clear, specific, and controlled. In the past 20 years, this theory and method have been widely used in system evaluation and control, data mining, group decision making, and other fields^31–36^. This paper takes the loess collapse of oil and gas pipelines as the research object. It proposes a comprehensive evaluation model of loess collapsibility based on standard cloud theory, which simplifies the preprocessing process of quantitative data and optimizes the cloud method and qualitative evaluation results. The results of cloud theory are compared with those of extension theory, PGRMS, and expert evaluation. The validity and rationality of the model are verified, and it provides a reference for the study of geological hazards in oil and gas pipelines.

## Methodology

### Construction of comprehensive evaluation system for pipeline loess collapsibility risk

The loess collapse disaster of oil and gas pipelines is the loess collapse of pipelines (including historical and potential) as the disaster-causing body, and the disaster-causing body of the geological disaster system, which takes the pipeline engineering body as the carrier, is under certain engineering geological conditions (topography, landform, tectonic activity, hydrogeology, stratigraphic lithology, etc.) and induced conditions (irrigation and precipitation). The disaster-bearing body is the main body of the pipeline project, including steel pipelines and anti-corrosion coatings, station valve chambers, etc., the laying of the channels (position, method, burial depth, etc.) in the loess-affected range, and the improvement of pipe trench soil. Filling, tamping, hydraulic protection, etc., directly affect the carrying method and carrying capacity of the pipeline. Besides, these complex factors together constitute the vulnerability factor of the risk assessment. And a series of prevention and control measures in pipeline maintenance (excavation, backfilling, tamping and filling hydraulic change measures, etc.) together constitute the vulnerability factors of risk assessment.

**Figure 1:**
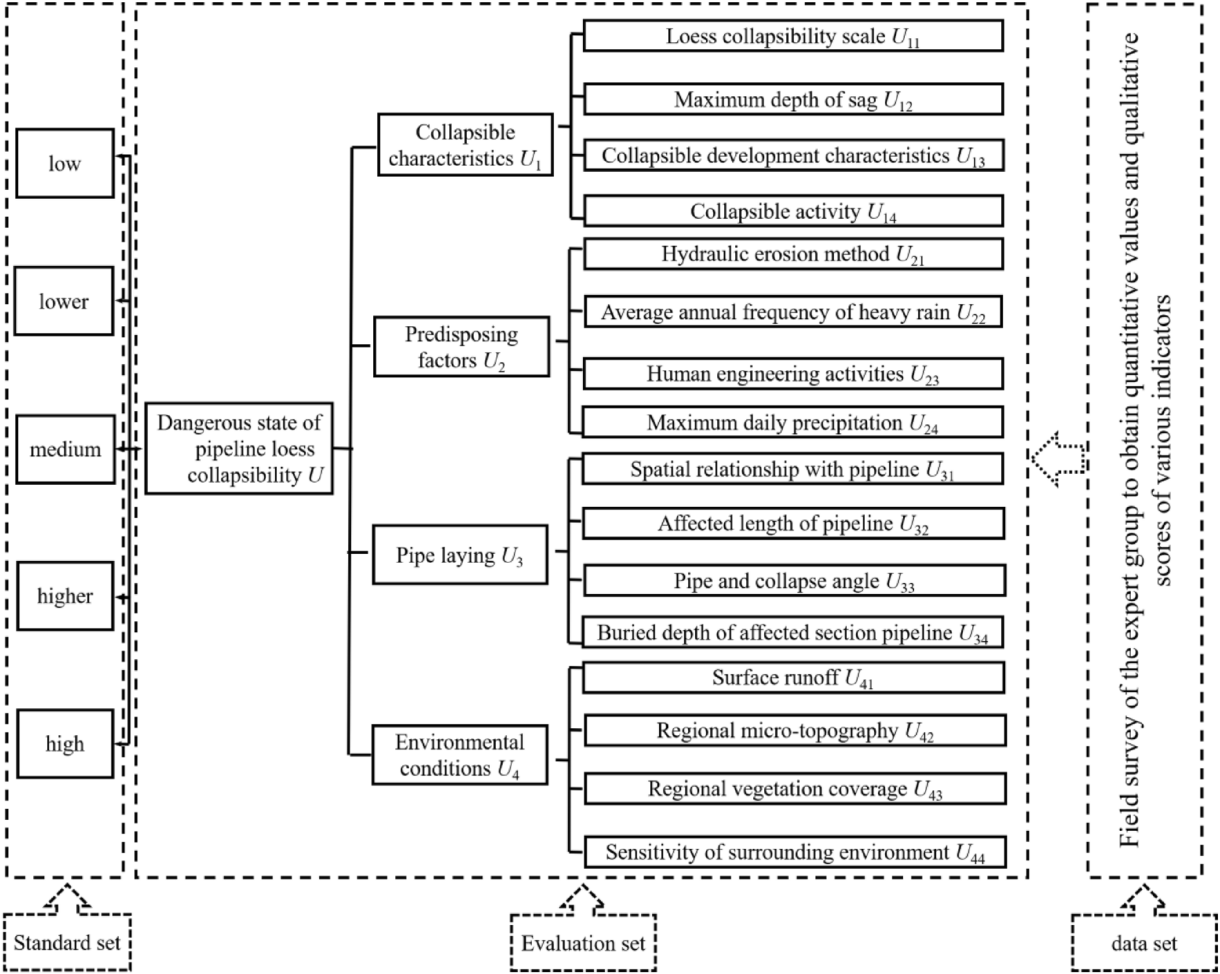
Pipeline loess collapsibility risk evaluation system diagram.

Based on the analysis of the constituent elements of the pipeline mentioned above, loess collapsibility hazard system, combined with related research results^5,15–18,25–29^, the independent representative adaptability of each indicator and the hierarchy of the evaluation system are considered and optimized and proposed a multi-level comprehensive evaluation index system consisting of four first-level indicators and sixteen second-level indicators. As shown in Fig. 1, there are eight quantitative indicators and eight qualitative indicators among the sixteen second-level indicators. The dimensions, level, attribute, etc., are different. How to organically integrate these complex indicators to participate in model calculations is the focus of evaluation research.

### Cloud theory

In a certain amount of universe U, there is X ⊆ U, and T is a qualitative concept in U space. When the value x (x ∈ X) is T on the membership C_T_ (x) ∈ [0,1], it is stable A random number with a tendency (such as a normal distribution), that is, there is formula (1),

$$ C_{T} \left( x \right):\forall x \in X\left( {X \subseteq U} \right)x \to C_{T} \left( x \right) $$

then the mapping of the concept T from the universe U to the interval [0,1] in the number domain space is called cloud^30^, and each x is a cloud drop; the digital feature C (Ex, En, He)^30^ is the control parameter of cloud distribution, where Ex is the central value of the universe, called expectation; En is the range of cloud droplet distribution that can be accepted by qualitative concepts in the universe space (that is, the degree of fuzzy picture), called entropy; He is the entropy of En, which is jointly determined by its randomness and ambiguity (that is, the degree of condensation of cloud drops in the universe of discourse, as shown in Fig. 3), and is called hyper-entropy. This paper introduces a one-dimensional regular cloud generator with universal applicability^32^, and its main algorithm is as follows^30–36^.

#### Forward cloud algorithm

1. generating a normal random number taking En as expectation and He as standard deviation, wherein norm is a function generating random numbers which obey normal distribution; $y_{i} = norm\left( {En,He^{2} } \right)$
2. generating a normal random number with Ex as an expected value and Y^2^ as a variance; $x_{i} = norm\left( {Ex,y^{2} } \right)$
3. Calculating the certainty of random number
  2 $$ \mu _{i}  = e^{{ - (x_{i}  - Ex)^{2} /(2y_{2}^{i} )}}  $$
4. output a cloud droplet; $\left( {x_{i} ,\mu_{i} } \right)$

And (5) repeating the steps (1) to (4) until the required N cloud droplets are generated, and the N cloud droplets form a cloud to realize the uncertainty cloudification of the concept.

#### Reverse cloud algorithm

(1) Sample mean calculated from cloud droplets Xi (cloud expectation)

3 $$ Ex = \overline{X} = \frac{1}{n}\sum\limits_{i = 1}^{n} {x_{i} } $$

(2) Calculating the Entropy of Cloud Droplet

4 $$ En = \sqrt {\frac{\pi }{2}} \times \frac{1}{n}\sum\limits_{i = 1}^{n} {\left| {x_{i} - Ex} \right|} $$

(3) Calculating the Excess Entropy of Cloud Droplet

5 $$ He = \sqrt {\left| {\frac{1}{n - 1}\sum\limits_{i = 1}^{n} {(x_{i} - \overline{X})^{2} } - En^{2} } \right|} $$

#### X-conditional cloud algorithm

1. the numerical characteristics (Ex, En, He) and the quantized value x of the concept are known, and a normal random number En' is generated by taking En as an expected value and He as a mean square deviation;
2. Calculate the degree of certainty y that the quantitative value X belongs to a certain concept, namely:
  6 $$ y = e^{{\frac{{ - (x - Ex)^{2} }}{{2(En )^{2} }}}} $$

#### Y-conditional cloud algorithm

1. Given the numerical characteristics (Ex, En, He) of the concept and the certainty y, y ∈ [0,1], a normal random number En′ is generated with En as the expected value and He as the mean square deviation;
2. calculating a quantitative value X satisfying the certainty y, namely:
  7 $$ x = Ex \pm En \times \sqrt { - 2\ln y} $$

#### Hybrid cloud algorithm

For two clouds C1 (Ex1, En1, He1) and C2 (Ex1, En1, He1) in the same universe, the mixed operation relation is (8)–(12), which can also be extended to n clouds.

8 $$ C_{1} + C_{2} = \left( {Ex_{1} + Ex_{2} ,\sqrt {En_{{_{1} }}^{2} + En_{{_{2} }}^{2} } ,\sqrt {He_{{_{1} }}^{2} + He_{{_{2} }}^{2} } } \right) $$

9 $$ C_{1} - C_{2} = \left( {Ex_{1} - Ex_{2} ,\sqrt {En_{{_{1} }}^{2} + En_{{_{2} }}^{2} } ,\sqrt {He_{{_{1} }}^{2} + He_{{_{2} }}^{2} } } \right) $$

10 $$ C_{1} \times C_{2} = \left( {Ex_{1} \times Ex_{2} ,\left| {Ex_{1} Ex_{2} } \right|\sqrt {\left( {\frac{{En_{1} }}{{Ex_{1} }}} \right)^{2} + \left( {\frac{{En_{2} }}{{Ex_{2} }}} \right)^{2} } ,\left| {Ex_{1} Ex_{2} } \right|\sqrt {\left( {\frac{{He_{1} }}{{Ex_{1} }}} \right)^{2} + \left( {\frac{{He_{2} }}{{Ex_{2} }}} \right)^{2} } } \right) $$

11 $$ C_{1} \div C_{2} = \left( {Ex_{1} \div Ex_{2} ,\left| {\frac{{Ex_{1} }}{{Ex_{2} }}} \right|\sqrt {\left( {\frac{{En_{1} }}{{Ex_{1} }}} \right)^{2} + \left( {\frac{{En_{2} }}{{Ex_{2} }}} \right)^{2} } ,\left| {\frac{{Ex_{1} }}{{Ex_{2} }}} \right|\sqrt {\left( {\frac{{He_{1} }}{{Ex_{1} }}} \right)^{2} + \left( {\frac{{He_{2} }}{{Ex_{2} }}} \right)^{2} } } \right) $$

### Comprehensive evaluation procedure for pipeline loess collapsibility risk

**Figure 2:**
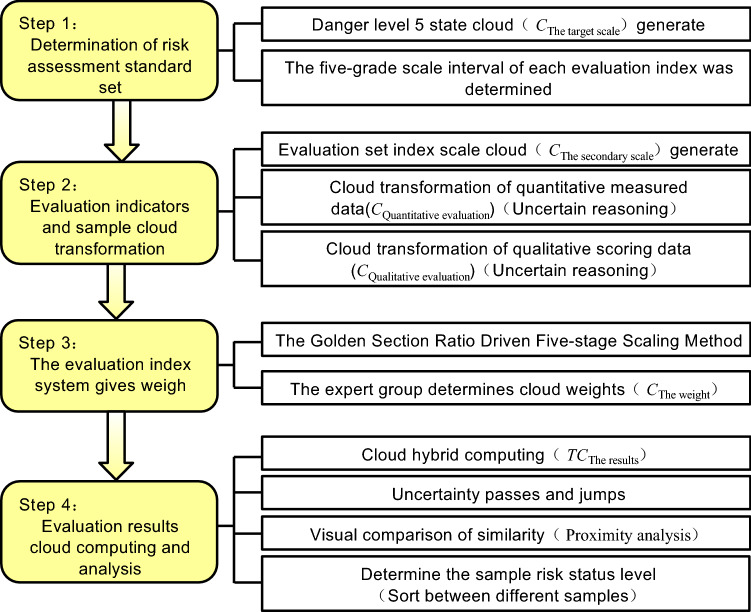
Flow chart of risk assessment based on cloud theory.

If the evaluation set layer (measured value + expert score value) in Fig. 1 can be mapped into a cloud with a specific distribution range and law, that is, a standard distribution cloud. In addition, it is assumed that the determination degree of each evaluation state in the common set layer is also subject to cloud distribution. Then, the comprehensive evaluation process of loess collapsibility risk of oil and gas pipelines based on cloud theory is simplified, as shown in Fig. 2. The specific steps are described as follows: related derivation and operation in the evaluation process and the driving of cloud generator are realized using MATLAB software.

*Step 1* Determination of risk state target cloud and evaluation index scale interval.

**Figure 3:**
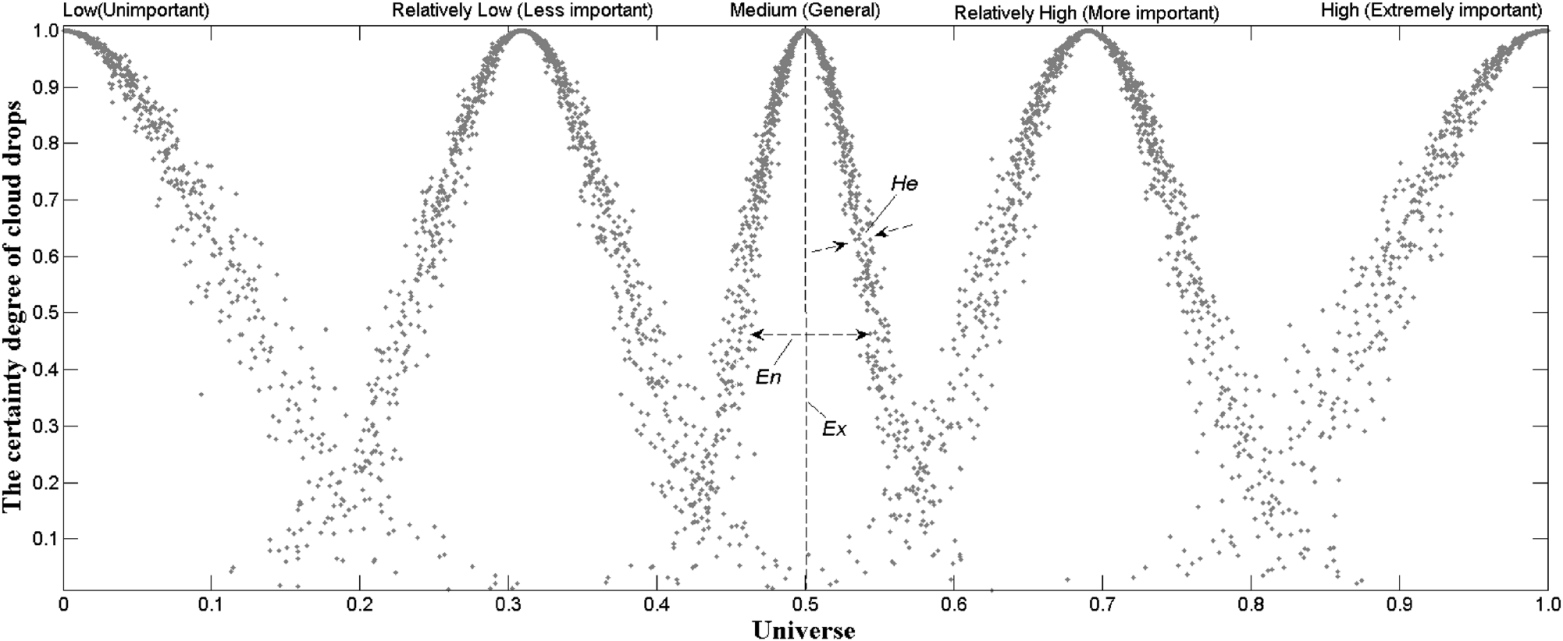
Cloud chart of evaluation criterion and weight.

To correspond to the results of the recommended method^2^ in the pipeline geological disaster risk management norms, this paper adopts 5-grade evaluations of high, relatively high, medium, relatively low, and low to scale the status of pipeline loess collapsibility risk as shown in Fig. 1 standard set. The golden ratio method^37^ is used to divide the language into five levels in the domain [0,1]. The cloud number characteristics and distribution of languages in each class are shown in Table 1 and Fig. 3. Similarly, the results of the 5-level evaluation interval division of 16 s-level indicators in the evaluation set are listed in Table 2. The eight quantitative indicators in Table 2 are divided into bilateral constrained approximations^32^. The eight qualitative indicators were scored and graded by experts on site^2,7,9,38^, and then clustered according to the cloud parameters of the corresponding level in Table 1.

**Table 1:** Digital parameters of cloud of evaluation criterion and weight.

Numeric Feature	Scale grade C scale/standard				
	Low (not important)	Relatively low (less important)	Medium (general)	Relatively high (more important)	High (very important)
*Ex*	0	0.309	0.5	0.691	1.0
*En*	0.1031	0.0640	0.0390	0.0640	0.1031
*He*	0.0130	0.0080	0.0050	0.0080	0.0130

**Table 2:** Evaluation criteria of each index.

	Index factor	Low	Relatively low	Medium	Relatively high	High
Collapsible characteristic U_1_	Collapse influence area U_11_	0–10 (5.000, 4.246, 0.010)	10–20 (15.000, 4.246, 0.010)	20–40 (30.000, 8.493, 0.010)	40–80 (60.000, 16.985, 0.010)	80–100 (90.000, 8.493, 0.010)
	Maximum collapse depth U_12_	0–0.4 (0.200, 0.170, 0.010)	0.4–0.7 (0.550, 0.127, 0.010)	0.7–1.0 (0.850, 0.127, 0.010)	1.0–1.5 (1.250, 0.212, 0.010)	1.5–3.0 (2.250, 0.637, 0.010)
	Characteristics of collapsible development U_13_	0–0.1 (0.050, 0.042, 0.010)	0.1–0.3 (0.200, 0.085, 0.010)	0.3–0.5 (0.400, 0.085, 0.010)	0.5–0.7 (0.600, 0.085, 0.010)	0.7–1 (0.850, 0.127, 0.010)
	Collapse activity U_14_	0–0.1 (0.050, 0.042, 0.010)	0.1–0.3 (0.200, 0.085, 0.010)	0.3–0.5 (0.400, 0.085, 0.010)	0.5–0.7 (0.600, 0.085, 0.010)	0.7–1 (0.850, 0.127, 0.010)
Inducing factor U_2_	Water erosion mode U_21_	0–0.1 (0.050, 0.042, 0.010)	0.1–0.3 (0.200, 0.085, 0.010)	0.3–0.5 (0.400, 0.085, 0.010)	0.5–0.7 (0.600, 0.085, 0.010)	0.7–1 (0.850, 0.127, 0.010)
	Rainstorm frequency U_22_	0–3 (1.500, 1.274, 0.010)	3–6 (4.500, 1.274, 0.010)	6–9 (7.500, 1.274, 0.010)	9–12 (10.500, 1.274, 0.010)	12–15 (13.500, 1.274, 0.010)
	Human engineering activity U_23_	0–0.1 (0.050, 0.042, 0.010)	0.1–0.3 (0.200, 0.085, 0.010)	0.3–0.5 (0.400, 0.085, 0.010)	0.5–0.7 (0.600, 0.085, 0.010)	0.7–1 (0.850, 0.127, 0.010)
	Multi-year maximum daily rainfall U_24_	0–10 (5.000, 4.246, 0.010)	10–30 (20.000, 8.493, 0.010)	30–50 (40.000, 8.493, 0.010)	50–70 (60.000, 8.493, 0.010)	70–100 (85.000, 12.739, 0.010)
Piping U_3_	Affected length of pipe U_31_	0–5 (2.500, 2.123, 0.010)	5–10 (7.500, 2.123, 0.010)	10–20 (15.000, 4.246, 0.010)	20–40 (30.000, 8.493, 0.010)	40–80 (60.000, 16.985, 0.010)
	Spatial relationship to piping U_32_	0–0.1 (0.050, 0.042, 0.010)	0.1–0.3 (0.200, 0.085, 0.010)	0.3–0.5 (0.400, 0.085, 0.010)	0.5–0.7 (0.600, 0.085, 0.010)	0.7–1 (0.850, 0.127, 0.010)
	Pipe collapse angle U_33_	0–20 (10.000, 8.493, 0.010)	20–40 (30.000, 8.493, 0.010)	40–60 (50.000, 8.493, 0.010)	60–80 (70.000, 8.493, 0.010)	80–90 (85.000, 4.246, 0.010)
	Buried depth of pipeline U_34_	0–0.8 (0.400, 0.340, 0.010)	0.8–1.4 (1.100, 0.255, 0.010)	1.4–2.0 (1.700, 0.255, 0.010)	2.0–2.5 (2.250, 0.212, 0.010)	2.5–6.0 (4.250, 1.486, 0.010)
Ambient conditions U_4_	Surface runoff U_41_	0–0.1 (0.050, 0.042, 0.010)	0.1–0.3 (0.200, 0.085, 0.010)	0.3–0.5 (0.400, 0.085, 0.010)	0.5–0.7 (0.600, 0.085, 0.010)	0.7–1 (0.850, 0.127, 0.010)
	Microgeomorphology U_42_	0–1 (0.500, 0.425, 0.010)	1–3 (2.000, 0.849, 0.010)	3–5 (4.000, 0.849, 0.010)	5–7 (6.000, 0.849, 0.010)	7–15 (11.000, 3.397, 0.010)
	Vegetation coverage U_43_	0–0.1 (0.050, 0.042, 0.010)	0.1–0.3 (0.200, 0.085, 0.010)	0.3–0.5 (0.400, 0.085, 0.010)	0.5–0.7 (0.600, 0.085, 0.010)	0.7–1 (0.850, 0.127, 0.010)
	Environmental sensitivity U_44_	0–1 (0.500, 0.425, 0.010)	1–3 (2.000, 0.849, 0.010)	3–5 (4.000, 0.849, 0.010)	5–7 (6.000, 0.849, 0.010)	7–15 (11.000, 3.397, 0.010)

*Step 2* Evaluate the indicators and the cloud transformation of samples to be evaluated.

For the eight quantitative indicators U_11_, U_12_, U_22_, U_24_, U_31_, U_33_, U_34_, and U_43_ in Table 3, there are comment levels with upper and relatively low boundary values of $x_{ij}^{1}$ and $x_{ij}^{2}$, and their normal cloud numerical characteristics can be expressed as:

12 $$ Ex_{ij} = \frac{{\left| {x_{ij}^{1} + x_{ij}^{2} } \right|}}{2} $$

**Table 3:** The environmental conditions and the development of loess collapsibility of the samples to be evaluated.

No	Collapsible area (m2)	Collapse Shape	Collapsible depth (m)	Vegetation Situation	Catchment condition	Microgeomorphology	Spatial relationship with pipes	Type of water damage	Influence length (m)	Harmful degree of pipelines	Buried depth of pipeline (m)	Human Engineering Activities
LC 1	70	Stripe	1.3	Apple	Dispersion	Flat Ground	Oblique Cross	Irrigation	40	Low	1.8	More
LC 2	44	Multilateral	1.9	Lily	Dispersion	Flat Bottom	Oblique Cross	Rainfall	10	relatively low	1.4	More
LC 3	33	Stripe	1.2	Corn	Catchment	Flat Ground	Vertical	Rainfall	3	relatively low	1.2	Moderate
LC 4	6	Stripe	2.5	Weed	Catchment	Flat Ground	Oblique Cross	Rainfall	12	Medium	1.5	More
LC 5	45	Stripe	0.4	Corn	Dispersion	Flat Ground	Oblique Cross	Irrigation	30	relatively low	1.5	Less
LC 6	50	Semicircle	1.7	Weed	Catchment	Scarp	Oblique Cross	Rainfall	10	High	2.2	More
LC 7	32	Ellipse	1.5	Wasteland	Dispersion	Flat Ground	Oblique Cross	Irrigation	10	relatively low	1.4	More
LC 8	50	Stripe	2.0	Potato	Catchment	Flat Ground	Oblique Cross	Irrigation	40	Medium	1.2	More
LC 9	6.3	Circle	2.5	Potato	Catchment	Slope	Oblique Cross	Rainfall	5	High	3.2	More
LC 10	14	Multilateral	0.2	Road	Catchment	Slope	Oblique Cross	Rainfall	5	relatively low	1.6	More

Since the boundary value refers to the existing research^6–8,12–19,26,31^ and expert suggestions, the indicators of continuous quantity description are subjectively divided according to specific standards and experience, but they are essentially vague. Therefore, the boundary needs to be softened so that the membership of the boundary value to the two adjacent state levels is equal, then $\exp \left[ { - \frac{{\left( {x_{ij}^{1} - x_{ij}^{2} } \right)^{2} }}{{8\left( {En_{ij} } \right)^{2} }}} \right] \approx 0.5$, and sorted out:

$$ En_{ij} = \frac{{\left| {x_{ij}^{1} x_{ij}^{2} } \right|}}{2.355} $$

From the cloud atomization ambiguity CD = 3He_ij_/En, He_ij_ generally takes a number between 0 and En/3 to satisfy the Gaussian cloud distribution^19^. Through many experiments in this paper, the practical value that is convenient for calculation is preferred. Generally, the larger the He_ij_, the thicker the normal cloud, and vice versa (Fig. 3).

**Figure 4:**
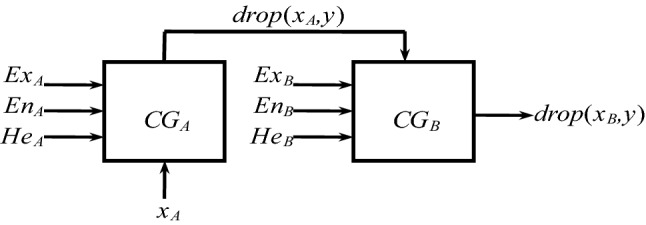
Single condition single rule generator.

Determine the level standardized cloud parameters of 8 quantitative indicators through formulas ⑿, ⒀, and multiple experiments, as shown in Table 3, and then build a single-condition single-rule cloud generator^23,24^ to realize the cloud conversion process of the measured value x_A_ of the quantitative index. The single-condition single-rule cloud generator is shown in Fig. 4. According to formulas ⑹ and ⑺, firstly generate a normal random number En_A_′ with En_A_ as the expected value and He_A_ as the mean square error, and calculate the certainty y = exp[-(x_A_-Ex_A_ )2/2(En_A_')2]; Regenerate the normal random number En_B_′ with En_B_ as the expected value and He_B_ as the mean square error. If the antecedent activates the rising or falling edge x_A_ ≤ Ex_A_ or x_A_ > Ex_A_, the latter is the same. Activate the value of rising or falling edge x_B_ = Ex_B_-En_B_′ × (− 2lny)0.5 or x_B_ = Ex_B_ + En_B_′ × (− 2lny)^0.5^.

The output results of the X-condition cloud and Y-condition cloud in the regular cloud generator are obtained through random processes. Therefore, for the same input, the output value received by the cloud inference method each time has uncertain characteristics. It fluctuates up and down within a reasonable range and has a stable tendency as a whole, obeying Gaussian distribution^21–24^. Through the cloudification of quantitative indicators: On the one hand, the subjective division of the level boundary is softened; on the other hand, x_A_ of different dimensions and magnitudes are calculated by X, Y-conditional cloud generators, and the data is dimensionless and normalized. This simplifies the pre-processing procedure. This method ensures the effective transmission and inheritance of uncertainty in the evaluation process, which is significantly better than other quantitative evaluation theories.

For the eight qualitative indicators of U_13_, U_14_, U_21_, U_23_, U_32_, U_41_, U_42_, and U_44_ in Table 3, it is impossible to use quantitative values to characterize the attributes accurately. This article is based on the scores of the expert group (10) members, whichever is Average^25^, score and evaluate eight qualitative indicators one by one; after obtaining the corresponding values, process them according to the process of cloudification of quantitative indicators and participate in the evaluation system.

### Evaluation results integrated cloud computing

*Step 3* Determination of the weight cloud of indicators at all levels of the evaluation system.

The weight reflects the relative importance and contribution rate of the various factors in the evaluation system to the goal and the key “hub” for the continuous transmission and rise of cloud theory. The risk evaluation indicators of loess collapsibility in oil and gas pipelines are primarily qualitative. It is difficult to directly obtain the weight value of each hand through objective weighting processes such as entropy method, coefficient of variation method, and gray correlation method; at the same time, to avoid analytic hierarchy process, etc. The shortcomings of personal empowerment are artificially arbitrary. This article invites seven industry experts and three technical backbones of pipeline operators to form an expert group to carry out the weighting of the evaluation system and the scoring of sample qualitative indicators. The language description of the relative importance of the participating indicators is not essential, second important, general, and more critical. And the significant 5-level state, based on the golden ratio-driven method, the index weights are assigned 5-level scale weights on the domain [0,1]. The corresponding cloud digital features and clouds are shown in Table 1 and Fig. 3, consistent with People's scientific understanding of things. Thus, while eliminating subjective arbitrariness, transforms qualitative language into weight values for calculation, reflecting the ambiguity, randomness, and complexity of the evaluation process.

*Step 4* Integrate the evaluation results into cloud computing.

Following the evaluation index system and cloud weight determination process of cloud conversion in the previous article, according to the hybrid cloud calculation formulas (8)–(11), the weighted average method is used to transfer the evaluation results of the bottom layer of the comment concentration to the upper layer, and then to the target layer, which is the risk of the evaluation object. The resulting cloud can be used for visual analysis and decision-making with the ruler cloud.

14 $$ TC = \sum\limits_{i = 1}^{n} {\omega_{i} C_{i} /\sum\limits_{i = 1}^{n} {\omega_{i} } } $$

In the formula, TC is the comprehensive cloud of the evaluation target, C_i_ and ω_i_ are the i-th index cloud and the weight cloud, respectively, and ω_i_C_i_ represents the multiplication of the two clouds. This process transfers uncertain reasoning and enables the concept of cloud theory to rise continuously.

## Analysis of engineering examples

Nearly 40% of the length of the Lan(zhou)-Cheng(du) Crude Oil Pipeline, a representative project of China's north–south oil transport, is laid in the Longxi Basin of the Loess Plateau, which is a typical collapsible loess distribution area. This area is located in the southwestern part of the Loess Plateau, bordered by the Qinba Mountains in the south and Wushaoling in the west. It is a continental temperate semi-arid climate zone with significant temperature differences and slight rainfall. The annual average temperature ranges from 4 to 14 ℃. Precipitation accounts for 50 to 70% of the yearly total, which is also a period when geological disasters frequently occur. Most of the land types in the area across which the pipeline is laid are agricultural land, and the primary vegetation is the cash crops. The vegetation coverage rate is generally at a medium level.

### Data sources

According to the 2020 pipeline geological disaster survey data, there are 109 geological disasters of various types in the Longxi Basin section of the Lancheng Pipeline, and there is 54 loess collapses, accounting for 49.54% of the total disasters. It can be seen that the loess collapses are densely developed and are prone to The characteristics of Gaofa. This paper randomly selects ten samples of risk assessment from 54 pipeline loess collapse disasters, and their specific locations are the pipeline mileage K7 + 110, K27 + 780, K32 + 720, K43 + 460, K43 + 920, K47 + 940, K57 + 900, K87 + 440, K168 + 850, K257 + 733 (numbered as LC 1–LC 10).

The essential characteristics of the selected loess collapsibility disasters are shown in Table 3. The initial data of the eight quantitative indicators of 10 samples to be evaluated are obtained from the existing meteorological, geographic, and geological data and on-site measurement. The results are listed in Table 4; the qualitative indicators are invited to the site according to the third step of the methodology. Judgement, record each expert's scoring value for different hands of each loess collapsibility and find the average value as the participating data^7,9,38^. Table 5 only lists the scores of 10 experts on the collapse disaster activity U14 in the sample indicators to be evaluated to simplify the length. The parameters of the remaining seven indicators can be obtained in the same way and participate in the evaluation system.

**Table 4:** Initial data of eight quantitative indexes.

Indicator name	LC 1	LC 2	LC 3	LC 4	LC 5	LC 6	LC 7	LC 8	LC 9	LC 10
Collapse influence area U_11_ (m2)	70	44	33	6	45	50	32	50	6.3	14
Maximum collapsible depth U_12_ (m)	1.3	1.9	1.2	2.5	0.4	1.7	1.5	2.0	2.5	0.2
Rainstorm frequency/year U_22_	10	9	9	10	10	13	9	10	8	13
Maximum average daily rainfall for many years U_24_ (mm)	75	72	75	80	70	80	80	72	70	80
Duct influence length U_31_ (m)	40	10	3	12	30	10	10	40	5	5
Pipe and Collapse Angle U_33_ (°)	12	16	86	12	81	72	21	15	45	72
Buried depth of pipeline U_34_ (m)	1.8	1.4	1.2	1.5	1.5	2.2	1.4	1.2	3.2	1.6
Vegetation coverage U_43_ (%)	0.95	0.43	0.12	0.33	0.47	0.18	0.06	0.34	0.21	0.02

**Table 5:** Qualitative indicator U14 expert score summary table.

Expert Serial Number	LC 1	LC 2	LC 3	LC 4	LC 5	LC 6	LC 7	LC 8	LC 9	LC 10
1	0.45	0.88	0.83	0.42	0.23	0.84	0.92	0.56	0.21	0.54
2	0.84	0.69	0.45	0.65	0.31	0.77	0.66	0.35	0.35	0.53
3	0.62	0.53	0.68	0.72	0.33	0.76	0.74	0.32	0.45	0.41
4	0.65	0.67	0.77	0.42	0.62	0.66	0.68	0.42	0.48	0.35
5	0.77	0.78	0.46	0.33	0.54	0.68	0.77	0.57	0.38	0.32
6	0.60	0.62	0.70	0.55	0.45	0.72	0.72	0.38	0.46	0.28
7	0.64	0.89	0.68	0.63	0.67	0.83	0.62	0.39	0.28	0.26
8	0.55	0.92	0.72	0.65	0.46	0.91	0.63	0.46	0.34	0.64
9	0.80	0.95	0.66	0.64	0.34	0.66	0.88	0.45	0.22	0.54
10	0.82	0.82	0.73	0.51	0.24	0.82	0.69	0.33	0.25	0.24
Average value	0.67	0.78	0.66	0.55	0.42	0.77	0.73	0.42	0.34	0.41

### Calculation of results

**Figure 5:**
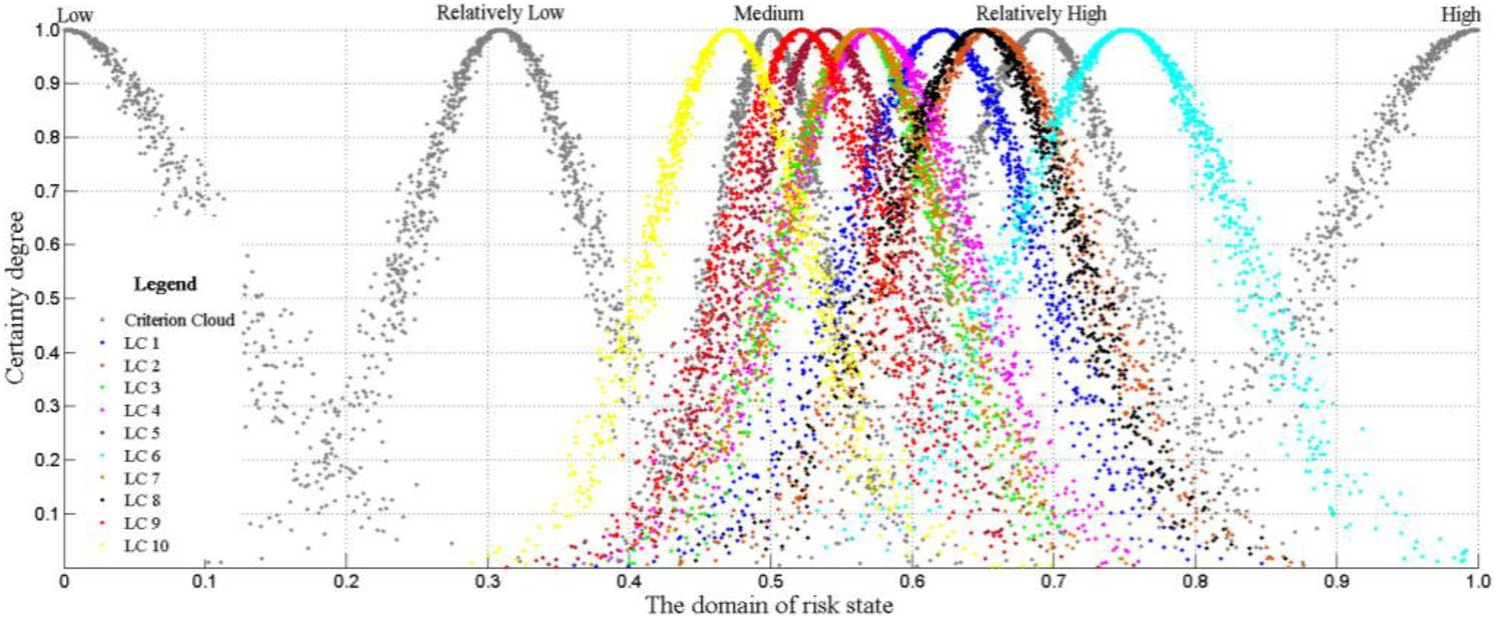
Cloud chart of loess collapsibility risk grade of pipeline.

First, use the single condition single rule cloud inference (1000 cycles of MATLAB generator) algorithm in the second step to calculate the cloud digital feature values of the 16 indicators in the 1–10 loess collapsibility, as shown in Table 6 Column. Then use the algorithm in formulas (8)–(11) to compare the index cloud C_evaluation_ and cloud C_weight_ in Table 6, according to the fourth step Chinese formula (14) upload the second-level index U_ij_ to the first-level index U_i_ and then pass it to the target layer U of the evaluation set. That is, the results of loess collapsibility risk in each sample area are TC_results_, which are TC_1_ (0.6210, 0.0670, 0.0073), TC_2_ (0.6552, 0.0679, 0.0075), TC_3_ (0.5646, 0.0572, 0.0068), TC_4_ (0.5733, 0.0665, 0.0067), TC_5_ (0.5394, 0.0568, 0.0060), TC_6_ (0.7517, 0.0797, 0.0085), TC_7_ (0.5648, 0.0589, 0.0065), TC_8_ (0.6473, 0.0679, 0.0076), TC_9_ (0.5220, 0.0539, 0.0067), TC_10_ ( 0.4708, 0.0551, 0.0058). Afterward, according to steps 1–5 of the forward cloud algorithm, the TC results were generated to generate the pipeline loess collapsibility danger cloud as shown in Fig. 5 superimposed the predetermined ruler cloud, and visualized comparison and closeness analysis were performed to determine the sample risk status level.

**Table 6:** Calculation results of cloud for each index level and weight.

Evaluation Index and cloud weight		Engineering samples and cloud eigenvalues				
		LC 1	LC 2	LC 3	LC 4	LC 5
Collapsible characteristic U_1_ (1, 0.1031, 0.013)	Collapse influence area U_11_ (1, 0.1031, 0.013)	(0.7286, 0.0046, 0.0007)	(0.5648, 0.0086, 0.0003)	(0.5138, 0.0018, 0.0002)	(0.0243, 0.0031, 0.0004)	(0.6345, 0.0067, 0.0008)
	Maximum collapse depth U_12_ (1, 0.1031, 0.013)	(0.7061, 0.002, 0.0002)	(0.9429, 0.0072, 0.0007)	(0.6758, 0.0019, 0.0001)	(1.0405, 0.0051, 0.0009)	(0.233, 0.0106, 0.0006)
	Characteristics of collapsible development U_13_ (0.5, 0.309, 0.005)	(0.7446, 0.0094, 0.001)	(0.9428, 0.0086, 0.0009)	(0.737, 0.008, 0.0016)	(0.6529, 0.0064, 0.0011)	(0.5093, 0.0016, 0.0002)
	Collapse activity U_14_ (0.309, 0.064, 0.008)	(0.5235, 0.0041, 0.001)	(0.6757, 0.0025, 0.0002)	(0.3242, 0.0026, 0.0003)	(0.7142, 0.0041, 0.0007)	(0.5189, 0.0034, 0.0001)
Inducing factor U_2_ (0.691, 0.064, 0.008)	Water erosion mode U_21_ (0.5, 0.309, 0.005)	(0.691, 0.064, 0.0001)	(0.7139, 0.0041, 0.0009)	(0.3696, 0.0104, 0.0009)	(0.5326, 0.0058, 0.0003)	(0.3695, 0.0099, 0.0024)
	Rainstorm frequency U_22_ (0.691, 0.064, 0.008)	(0.6658, 0.003, 0.0003)	(0.5459, 0.0058, 0.0005)	(0.5461, 0.0062, 0.0004)	(0.6658, 0.0031, 0.0001)	(0.6661, 0.0033, 0.0005)
	Human engineering activity U_23_ (0.691, 0.064, 0.008)	(0.7292, 0.0063, 0.0017)	(0.6756, 0.0027, 0.0005)	(0.486, 0.0024, 0.0003)	(0.9592, 0.0061, 0.0004)	(0.3776, 0.0124, 0.0011)
	Maximum daily rainfall U_24_ (1, 0.1031, 0.013)	(0.9185, 0.0101, 0.0014)	(0.7814, 0.0106, 0.0005)	(0.9196, 0.0101, 0.0004)	(0.9593, 0.0051, 0.0005)	(0.7664, 0.0093, 0.0006)
Piping U_3_ (0.5, 0.309, 0.005)	Duct influence Length U_31_ (0.691, 0.064, 0.008)	(0.7662, 0.0097, 0.0003)	(0.384, 0.0094, 0.0015)	(0.0242, 0.0031, 0.0002)	(0.4725, 0.0036, 0.0002)	(0.691, 0.0001, 0.0001)
	Spatial relationship to piping U_32_ (1, 0.1031, 0.013)	(0.3469, 0.0064, 0.0009)	(0.9589, 0.0062, 0.0004)	(0.9755, 0.0035, 0.0005)	(0.3397, 0.0051, 0.0005)	(0.6604, 0.0051, 0.0005)
	Pipe collapse angle U_33_ (0.309, 0.064, 0.008)	(0.0242, 0.003, 0.0004)	(0.0727, 0.0087, 0.001)	(1.0244, 0.0031, 0.0004)	(0.0244, 0.003, 0.0003)	(0.9029, 0.0121, 0.0022)
	Buried depth of pipeline U_34_ (0.691, 0.064, 0.008)	(0.5152, 0.0021, 0.0003)	(0.3847, 0.0099, 0.0008)	(0.3342, 0.0032, 0.0002)	(0.4694, 0.0042, 0.0006)	(0.4692, 0.0043, 0.0003)
Ambient conditions U_4_ (0.309, 0.064, 0.008)	Surface runoff U_41_ (0.691, 0.064, 0.008)	(0.4628, 0.0065, 0.001)	(0.6604, 0.0055, 0.0012)	(0.5138, 0.0024, 0.0002)	(0.3243, 0.0027, 0.0001)	(0.2863, 0.0037, 0.0009)
	Microgeomorphology U_42_ (0.309, 0.064, 0.008)	(0.039, 0.0048, 0.0004)	(0.3467, 0.0046, 0.0004)	(0.4723, 0.0035, 0.0005)	(0.6759, 0.0019, 0.0002)	(0.3544, 0.0059, 0.0002)
	Vegetation coverage U_43_ (0.309, 0.064, 0.008)	(1.0819, 0.0118, 0.0013)	(0.514, 0.0024, 0.0005)	(0.1825, 0.0553, 0.026)	(0.4675, 0.0057, 0.0006)	(0.5327, 0.0057, 0.001)
	Environmental sensitivity U_44_ (0.5, 0.309, 0.005)	(0.4771, 0.0031, 0.0002)	(0.4863, 0.0017, 0.0002)	(0.4815, 0.0024, 0.0004)	(0.9331, 0.0084, 0.0006)	(0.7514, 0.0078, 0.001)

Evaluation index and cloud weight		Engineering samples and cloud eigenvalues				
		LC 6	LC 7	LC 8	LC 9	LC 10
Collapsible characteristic U_1_ (1, 0.1031, 0.013)	Collapse influence area U_11_ (1, 0.1031, 0.013)	(0.6532, 0.0048, 0.0006)	(0.5092, 0.0011, 0.0001)	(0.6533, 0.0047, 0.0008)	(0.0315, 0.0037, 0.0004)	(0.2939, 0.0018, 0.0002)
	Maximum collapse depth U_12_ (1, 0.1031, 0.013)	(0.8267, 0.0177, 0.0022)	(0.7663, 0.01, 0.0012)	(0.9595, 0.0051, 0.0008)	(1.0405, 0.0053, 0.0004)	(0, 0.1031, 0.013)
	Characteristics of collapsible development U_13_ (0.5, 0.309, 0.005)	(0.9344, 0.0094, 0.0015)	(0.9019, 0.0152, 0.0017)	(0.5092, 0.0017, 0.0005)	(0.4722, 0.005, 0.0007)	(0.5046, 0.0008, 0.0001)
	Status of disaster activities U_14_ (0.309, 0.064, 0.008)	(0.9019, 0.0147, 0.0005)	(0.3779, 0.0116, 0.0017)	(0.7369, 0.0084, 0.0014)	(0.7141, 0.0041, 0.0001)	(0.2861, 0.0039, 0.0006)
Inducing factor U_2_ (0.691, 0.064, 0.008)	Water erosion mode U_21_ (0.5, 0.309, 0.005)	(0.9021, 0.0146, 0.0014)	(0.332, 0.0041, 0.0007)	(0.4674, 0.0058, 0.0015)	(0.3166, 0.0013, 0.0002)	(0.4675, 0.0056, 0.0005)
	Rainstorm frequency U_22_ (0.691, 0.064, 0.008)	(0.9597, 0.0052, 0.0009)	(0.5461, 0.0058, 0.0012)	(0.6657, 0.0032, 0.0005)	(0.5153, 0.0019, 0.0001)	(0.9595, 0.0051, 0.0003)
	Human engineering activity U_23_ (0.691, 0.064, 0.008)	(0.7292, 0.0066, 0.0004)	(0.7373, 0.0084, 0.002)	(0.6757, 0.0025, 0.0005)	(0.9022, 0.015, 0.0016)	(0.7521, 0.0112, 0.0021)
	Maximum daily rainfall U_24_ (1, 0.1031, 0.013)	(0.9597, 0.0053, 0.0009)	(0.9593, 0.005, 0.0006)	(0.782, 0.011, 0.0007)	(0.7658, 0.0096, 0.0004)	(0.9593, 0.0051, 0.0003)
Piping U_3_ (0.5, 0.309, 0.005)	Duct Influence length U_31_ (0.691, 0.064, 0.008)	(0.3844, 0.0097, 0.0017)	(0.3844, 0.0093, 0.0008)	(0.7664, 0.0094, 0.001)	(0.2333, 0.0097, 0.0011)	(0.2338, 0.0094, 0.001)
	Spatial relationship to piping U_32_ (1, 0.1031, 0.013)	(0.752, 0.0099, 0.0011)	(0.4815, 0.0032, 0.0006)	(0.7833, 0.0159, 0.0037)	(0.1836, 0.0545, 0.0294)	(0.5094, 0.0017, 0.0003)
	Pipe collapse angle U_33_ (0.309, 0.064, 0.008)	(0.7061, 0.0018, 0.0002)	(0.2414, 0.0087, 0.0003)	(0.0604, 0.0076, 0.0012)	(0.4771, 0.003, 0.0001)	(0.6925, 0.0019, 0.0001)
	Buried depth of pipeline U_34_ (0.691, 0.064, 0.008)	(0.6757, 0.0021, 0.0003)	(0.3844, 0.0099, 0.0005)	(0.3342, 0.0032, 0.0005)	(0.9791, 0.0382, 0.004)	(0.4847, 0.0021, 0.0002)
Ambient conditions U_4_ (0.309, 0.064, 0.008)	Surface runoff U_41_ (0.691, 0.064, 0.008)	(1.0163, 0.0024, 0.0002)	(0.7524, 0.0108, 0.0018)	(0.472, 0.005, 0.0012)	(0.5278, 0.005, 0.0006)	(0.4907, 0.0016, 0.0001)
	Microgeomorphology U_42_ (0.309, 0.064, 0.008)	(0.2939, 0.0019, 0.0001)	(0.2567, 0.0069, 0.001)	(0.4589, 0.0054, 0.001)	(0.6305, 0.0074, 0.0016)	(0.843, 0.0193, 0.0028)
	Vegetation coverage U_43_ (0.309, 0.064, 0.008)	(0.2937, 0.0027, 0.0005)	(0.0246, 0.0079, 0.0045)	(0.4718, 0.0052, 0.001)	(0.3166, 0.0014, 0.0002)	(0.0801, 0.025, 0.017)
	Environmental sensitivity U_44_ (0.5, 0.309, 0.005)	(0.7136, 0.0028, 0.0002)	(0.4862, 0.0018, 0.0002)	(0.6685, 0.003, 0.0005)	(0.2563, 0.0067, 0.0008)	(0.5092, 0.0012, 0.0002)

Figure 5 shows that the risk result cloud of loess collapsibility of No. 1–10 which are basically similar to the ruler cloud in the form of the “skeleton”, are distributed between the “relatively low” and “relatively high” cloud rulers, and mainly concentrated in Between “medium” and “relatively high” cloud ruler. The specific manifestations are as follows: LC1 cloud is inclined to “relatively high cloud”; LC2 cloud is close to “relatively high cloud”; LC3 cloud is inclined to “ medium cloud”; LC4 cloud is close to “ medium cloud” and LC5 cloud is near “ medium cloud”; LC6 cloud is adjacent to “relatively high cloud”; LC7 cloud is adjacent to “ medium cloud”; LC8 is close to “relatively high cloud”; LC9 cloud is close to “ medium cloud”; LC10 cloud is close to “medium cloud”. The hazard degree of loess collapsibility to oil and gas pipelines is in the order of LC6 > LC2 > LC8 > LC1 > LC4 > LC2 > LC3 > LC5 > LC9 > LC10.

### Result analysis

To test the applicability and effectiveness of cloud theory in the evaluation of loess collapsibility risk of oil and gas pipelines, the extension theory method (a method based on fuzzy mathematics)^39–41^, PGRMS method^38^(a semi-quantitative way recommended by the code^2^, which is automatically realized by the pipeline geological disaster risk management system provided by China Petroleum Pipeline Science and Technology Research Center) and expert qualitative assessment method are used to carry out a risk assessment on ten loess subsidence sites respectively. The results are listed in Table 7.

**Table 7:** Comparison of risk assessment results of the No. 1–10 loess collapsibility of pipeline.

Sample to be evaluated	Normative method result	Extension theory method	Expert qualitative assessment method	Results of this paper
LC 1	High (0.2127)	Relatively high	Medium	Medium–high, bias high
LC 2	Relatively high (0.1973)	Medium	Medium	Medium–high, bias high
LC 3	Medium (0.677)	Medium	Relatively high	Medium–relatively high, incline to medium cloud
LC 4	Relatively low (0.0472)	Low	Low	Medium–relatively high, near medium cloud
LC 5	Medium (0.0513)	Relatively high	Relatively high	Medium–relatively high, near medium cloud
LC 6	Relatively high (0.1846)	High	Relatively high	Relatively high–high, next to relatively high
LC 7	Medium (0.0926)	Medium	Medium	Medium–relatively high, near medium cloud
LC 8	Relatively high (0.1773)	High	Relatively high	Medium–relatively high, next to relatively high
LC 9	Medium (0.0635)	Medium	Relatively low	Medium–relatively high, next to medium cloud
LC 10	Relatively low (0.0391)	Medium	Relatively low	Medium–relatively high, near medium cloud

Combining Table 7 and Fig. 5, we can see that the risk of loess collapsibility of No. 1–10 using the PGRMS method and extension theory method is consistent with the results of the method proposed in this paper, indicating the risk of loess collapsibility of oil and gas pipelines based on cloud theory The comprehensive evaluation method is practical and feasible, and can fully reflect the fuzziness and randomness of the participating variables in the evaluation process, which is more in line with people's thinking mode and expression habits on the understanding of the dangerous development of pipeline loess collapsibility, and also enables the evaluation results The expression of is visualized in the form of cloud drop distribution.

The results of this method were compared with those of the other three methods. The results showed that: (1) The consistency ratio of the evaluation results between the cloud theory method and the PGRMS method was 70%, the relatively high proportion was 20%, and the relatively low proportion was 10%. (2) The consistency ratio of the evaluation results between the cloud theory method and the extension theory method is 60%, the high proportion is 20%, and the low proportion is 20%. (3) The consistency ratio of cloud theory method and expert qualitative method is 30%, the high proportion is 50%, the low proportion is 20%; (4) The consistency ratio between PGRMS and expert qualitative method is 40%, with high proportion 40% and low proportion 20%. (5) The consistency ratio between PGRMS method and the extension theory method is 40%, 50% relatively high, and 10% relatively low. (6) The results of the extension theory method and the expert qualitative method are consistent with the proportion of 40%, the high proportion of 40%, the low proportion of 20%.

In summary, it is found that the cloud theory method introduced in this paper has a relatively high overall risk level for the pipeline loess collapsibility risk assessment result than the other three methods, which is more consistent with the actual situation of the example samples and is more conducive to the safety guarantee of pipeline operators in the study area. The evaluation results of the cloud theory method have high consistency with those of the PGRMS method and extension theory method but poor consistency with the evaluation results of the expert qualitative method. This is mainly because the cloud theory method, PGRMS method, and extension theory method all carry out quantitative processing on comprehensive indicators and carry out fusion operation through a particular mathematical model, which weakens subjective arbitrariness to a certain extent, and the factors involved in the evaluation are more comprehensive, and the evaluation process is systematic. However, the evaluation results of the expert qualitative method only rely on the macroscopic grading judgment of the expert group according to the individual experience. In the process, the subjective arbitrariness is strong. It is easy to be interfered with by different factors such as personal preference, professional background, comprehensiveness, and degree of understanding of various experts. As a result, the accuracy of the results is generally poor.

## Conclusion

1. The risk evaluation system of oil and gas pipeline loess collapsibility comprises a multi-level multi-objective complex index system. Common evaluation theories and methods cannot deal with the ambiguity and randomness of the system at the same time. Based on the analysis of the fuzziness, randomness, dimension, and magnitude difference of pipeline loess collapsibility evaluation indicators, this paper proposes a comprehensive evaluation model of the single loess collapsibility risk of oil and gas pipelines cloud theory. Through the risk assessment and verification analysis of 10 loess collapsible disaster points in the Lancheng Crude Oil Pipeline, its practicability and effectiveness are shown.

(2) The quantitative data in the evaluation comes from actual measurement and background data, and the qualitative information comes from the scoring and assignment of the expert group. The initial data source is relatively objective and reliable; the index weight cloud is determined by the 5-level golden ratio method, which is transmitted. The effective weighting of quantitative and qualitative indicators is realized based on system fuzziness and randomness.

(3) The single-condition-single-rule cloud conversion method is adopted for the quantitative indicators in the evaluation, which effectively transmits and inherits the ambiguity and randomness of the system, weakens the subjective division of hierarchical boundaries, and simplifies the pre-processing of initial data; for qualitative indicators After scoring and averaging the expert group, the single-condition-single-rule cloud conversion method is adopted, and the interference of subjective factors can be reduced to a certain extent by weakening the division of the grade boundary.
(4) The evaluation result is a cloud composed of three parameters: expected value, entropy, and super-entropy, which realizes the evaluation process of quantitative and qualitative integration, integrated decision-making, and the visualization of the final result is a complex system and is used to prevent the loess collapsibility disaster of oil and gas pipelines. Governance provides scientific and technological support and also proposes an effective new method for related research.

## References

[1] Girgin, S., & Krausmann, E. Analysis of pipeline accidents induced by natural hazards: final report. *Eur. Union: ResearchGate* (2016).

[2] National Energy Administration, People's Republic of China. SY/T 6828–2017, Technical Specification for Geological Hazards Risk Management of Oil and Gas Pipeline. Beijing: Petroleum Industry Press Ltd (2017).

[3] Girgin S, Krausmann E (2016). Historical analysis of U.S. onshore hazardous liquid pipeline accidents triggered by natural hazards. J. Loss Prevent Proc..

[4] Wang Y, Zhou L (1999). Spatial distribution and mechanism of geological hazards along the oil pipeline planned in western China. Eng. Geol..

[5] Fu P, Liu G, Liu C (2010). The study on the loess sinkhole hazard along a long-distance pipeline in the collapsible loess area. West-China Explor. Eng..

[6] Huang W, Zheng H, Li M (2019). Development history and prospect of oil & gas and transportation industry in China. Oil Gas Storage Transport..

[7] Kimiya Z, Fuzhan N (2020). A review of failure prediction models for oil and gas pipelines. J. Pipeline Syst. Eng. Pract..

[8] Wang X, Duan Q (2019). Improved AHP-TOPSIS model for the comprehensive risk evaluation of oil and gas pipelines. Petrol. Sci..

[9] Tong S., Wu Z. & Wang R. *et al.* Risk study on long-distance oil and gas pipelines engineering. Proceedings of the 5th International Conference on Electrical Engineering and Automatic Control. 583–590 (2016).

[10] Jamshidi A, Yazdani-Chamzini A, Yakhchali SH (2013). Developing a new fuzzy inference system for pipeline risk assessment. J. Loss Prevent. Proc..

[11] Fayaz M, Ahmad S, Hang L (2019). Water supply pipeline risk index assessment based on cohesive hierarchical fuzzy inference system. Proces.

[12] Wang P, Xu Z, Bai M (2012). Landslide risk assessment expert system along the oil and gas pipeline routes. Adv. Mater. Res..

[13] Vasseghi A, Haghshenas E, Soroushian A (2021). Failure analysis of a natural gas pipeline subjected to landslide. Eng. Fail. Anal..

[14] Teng M, Ke S (2021). Disaster impact assessment of the underground hazardous materials pipeline. J. Loss Prevent. Proc..

[15] Peng J, Sun P, Ogbonnaya I (2018). Loess caves, a special kind of geo-hazard on loess plateau, northwestern China. Eng. Geol..

[16] Wang J, Xiang W (2010). Situation and prevention of loess water erosion problem along the west-to-east gas pipeline in China. J. Earth Sci..

[17] Gao, Q. Research on characteristics of water damage along oil and gas pipeline on collapsible soil site. Lanzhou: Master’s Degree Thesis of Lanzhou University of Technology (2016).

[18] Guo, C. Study on comprehensive risk assessment and early-warning of geological disasters for Shan-Jing pipeline. Beijing: Doctor’s Degree Thesis of China University of Petroleum (2016).

[19] Jungsup U, Robert W (1996). Pipeline construction and reinstatement monitoring: current practice, limitations and the value of airborne videography. Sci. Total Environ..

[20] Liu J, Zhu D, Wang R (2018). Analysis on the settlement of long distance direct-buried pipeline under pipe-soil coupling effect. Oil-Gasfield Surf. Eng..

[21] Wang T, Yan X, Yang X (2010). Force analysis of suspended pipeline in collapsible loess based on elastic-plastic foundation model. J. China U Petrol..

[22] Xu L, Liu X, Chen F (2018). Mechanical analysis of buried suspended pipeline under the action of collapse. Eng. Mech..

[23] Zhang P, Long H, Li Z (2017). Finite element simulation on mechanical behavior of buried oil/gas pipeline in loess collapse process. J. Saf. Sci. Technol..

[24] Sarvanis GC, Spyros AK (2017). Analytical model for the strain analysis of continuous buried pipelines in geo-hazard areas. Eng. Struct..

[25] Yu Y, Yuan N, Yang P (2019). Application of fuzzy mathematics in damage assessment of loess collapsibility in long-distance pipeline. Chin. Overseas Archit..

[26] Gao Y, Luo Z, Bi A (2020). Risk assessment of coupling coordination of long-distance pipelines in collapsible loess region. J. Catastrophol..

[27] Yang, D. Study on risk assessment technology of loess collapse hazard of pipeline. Chengdu: Master’s Degree Thesis of Southwest Petroleum University (2014).

[28] Zhou, X. Loess collapse hazard fuzzy comprehensive evaluation technique for oil and gas pipeline. Lanzhou: Master’s Degree Thesis of Lanzhou University of Technology (2016).

[29] Li P, Sai V, Li T (2016). Review of collapse triggering mechanism of collapsible soils due to wetting. J. Rock Mech. Geotech. Eng..

[30] Li D, Du Y (2014). Artificial Intelligence with Uncertainty.

[31] Li S, Wang Z, Lai C (2020). Quantitative assessment of the relative impacts of climate change and human activity on flood susceptibility based on a cloud model. J. Hydrol..

[32] Fu HL, Huang Z, Huang HW (2017). Health diagnosis method of shield tunnel structure based on cloud theory. Chin. J. Eng..

[33] Li W, Wang Y, Du J (2017). Synergistic integration of graph-cut and cloud model strategies for image segmentation. Neurocomputing.

[34] Guo Y, Meng X, Meng T (2016). A novel method of risk assessment based on cloud inference for natural gas pipelines. J. Nat. Gas Sci. Eng..

[35] Zhao D, Li C, Wang Q (2020). Comprehensive evaluation of national electric power development based on cloud model and entropy method and TOPSIS: a case study in country. J. Clean. Prod..

[36] Yan F, Xu K (2019). Methodology and case study of quantitative preliminary hazard analysis based on cloud model. J. Loss Prevent. Proc..

[37] Wang J, Xiao W, Wang S (2010). An improved effectiveness evaluation method based on cloud model. Fire Control Command Control.

[38] Jing H, Hao J, Chen Y (2011). Technique and application of geological hazard risk semi-quantitative assessment of pipeline. Oil Gas Storage Transport..

[39] Guo Q, Shohel A, Hao Q (2020). Resilience assessment of safety system at subway construction sites applying analytic network process and extension cloud models. Reliab. Eng. Syst. Safe..

[40] Sun Z, Wang S, Zhang M (2015). Waterlogging risk assessment of pipeline based on extenics theory. Sci. Technol. Eng..

[41] Du Y, Zhang Y, Wu G (2020). Decision-making method of heavy-duty machine tool remanufacturing based on AHP-entropy weight and extension theory. J. Clean. Prod..

